# Parasitological and biochemical studies on cutaneous leishmaniasis in Shara’b District, Taiz, Yemen

**DOI:** 10.1186/s12941-017-0224-y

**Published:** 2017-07-04

**Authors:** Qhtan Asmaa, Salwa AL-Shamerii, Mohammed Al-Tag, Adam AL-Shamerii, Yiping Li, Bashir H. Osman

**Affiliations:** 1 College of Environment, Hohai University, Nanjing, 210098 China; 2 Faculty of Medical Science, Taiz University, Taiz, Yemen; 3 Department of Applied Microbiology, Taiz University, Taiz, Yemen; 4 Faculty of Applied Science, Direction of Scientific Research, Taiz University, Taiz, Yemen; 5 College of Engineering, Sinnar University, Sinnar, Sudan

**Keywords:** Leishmaniasis, Prevalence, Malondialdehyde, Free radicals scavengers, Yemen

## Abstract

**Background:**

The leishmaniasis is a group of diseases caused by intracellular haemoflagellate protozoan parasites of the genus Leishmania. Leishmaniasis has diverse clinical manifestations; *cutaneous leishmaniasis* (CL) is the most common form of leishmaniasis which is responsible for 60% of disability-adjusted life years. CL is endemic in Yemen. In Shara’b there is no reference study available to identify the prevalence of endemic diseases and no investigation has been conducted for diagnosing the diseases.

**Methods:**

This study was conducted in villages for CL which collected randomly. The study aimed at investigating the epidemiological factors of CL in Shara’b by using questioner. Symptoms of lesions in patients suffering from CL, confirmed by laboratory tests, gave a new evidence of biochemical diagnosis in 525 villagers aged between 1 and 60 years old. Venous bloods were collected from 99 patients as well as from 51 control after an overnight fast.

**Results:**

The percentage prevalence of CL was found 18.8%. The prevalence rate of infection among males (19.3%) was higher than females (18.40%). Younger age group (1–15) had a higher prevalence rate (20.3%) than the other age groups. Furthermore, the population with no formal education had the higher rate of infection (61% of the total). A significant increase of serum malondialdehyde (P < 0.001) in CL patients was obtained. The highest level of MDA may be due to over production of ROS and RNS results in oxidative stress and the acceleration of lipid peroxidation in CL patients.

**Conclusions:**

There were high prevalence rates of CL in Shara’b. The patient who had CL has been found with many changes in some biochemical levels. This study provides a clear indication on the role of MDA as an early biochemical marker of peroxidation damage occurring during CL. Increased uric acid, and catalase activity was provided of free radical.

## Background

Leishmaniasis is a diseases caused by obligatory and intracellular haemoflagellate protozoan parasites of the genus *Leishmania* (family trypanosomatidae). Human leishmaniasis is a compound disease with numerous clinical forms, which variety from mild self-healing cutaneous lesions to fatal visceral disease and neotropics [1]. It is overwhelmingly referred to as a group of diseases in view of the fact that the varied spectrum of clinical manifestations, which has the scope from small cutaneous nodules to overall mucosal tissue destruction. CL can be caused by a number of *Leishmania* spp. and is transferred to human beings and animals by sandflies. Cutaneous leishmaniasis is predominant in 88 countries including 77 of developing, tens of millions of people are at hazard of getting this disease and it is estimated that each year 1–1.5 million new cases appear. CL was endemic in Yemen [2, 22]. It has been recognized as a public health problem predominated by infection with the highest burden of leishmaniasis, but has not been fully documented. Cutaneous leishmaniasis is endemic and most of the cases are registered in Lahg, Abun, Hagga and Sa’adah Taiz Governorates [3].

CL is transmitted by the bite of an infected sand fly. When the parasites enter the Polymorph nuclear neutrophils (PML) and the monocyte macrophage cells play an important role in the host defense [4]. These cells are capable to generating a large amounts of extremely toxic molecules, such as reactive oxygen species (ROS), comprise superoxide radicals (O_2_^−^), hydrogen peroxide (H_2_O_2_) and hydroxyl radicals (OH), and reactive nitrogen species (RNS), inclusive nitric oxide (NO) Bogdan C Rolling off Bacteria, parasites and tumor cells motivate macrophages to synthesize considerable amounts of NO which has cytotoxic effects on these activators.

ROS and RNS are capable of degrading many biomolecules, including DNA, carbohydrates and proteins. Furthermore, ROS and RNS can assault the polyunsaturated fatty acids of membrane lipids causing lipid peroxidation and the disorder of cell construction and function [5]. Lipid peroxidation is a well-recognized mechanism of cellular injury and is used as a marker of oxidative stress in cells and tissues [6].

Polyunsaturated fatty acid derived that are not stable, can decay hence forming many series of complex products [7]. They are degraded such as carbonyl compound which are plentiful Malondialdehyde (MDA) that is widely used as marker of lipid peroxidation [8]. High levels of lipid peroxidation products are accompanying with a variety of chronic diseases with parasitic infections [9]. The serum concentration of MDA was dignified in humans with cutaneous leishmaniasis to establish its connection in the pathological mechanism of the disease [10].

To avoid potential oxidative damage there are defense mechanisms systems which classified as enzymatic [superoxide dismutase (SOD), catalase, glutathione peroxidase (GSH peroxidase), glutathione reductase and GSH reductase] and non-enzymatic (vitamins and uric acid). The estimation of MDA level and antioxidant enzyme activity are the main standards in relation to the severity of probable peroxidation, which occur in the cell membrane [11]. Anti-oxidant vitamins for instance E, C, and A protect the cells from destruction in contradiction of free oxygen radicals generated consequently of parasites.

Antioxidant systems including vitamins have a cellular protective action against oxidative stress subsequent in cell, organ, and tissue damage because of parasitic invasion [12]. This study aimed to determine the prevalence of CL in some villages in Shara’b district, Taiz, Yemen, to investigate the risk factors that increase the prevalence of CL, and explore the evidence of free radicals and antioxidants during CL.

## Methods

### Study area

Shara’b is a district with an area of about 61,700 km^2^ and population of about 393, 425, forms about 12,000 km^2^ faraway from Taiz Governorate. Shara’b is divided into two districts: Shara’b Salam district with an area of 20,000 km^2^ and population of 146,650, and Shara’b Ar Rawnah district with area of 41,700 km^2^ and population about 18, 6955. Shara’b district has mountains and Aqueducts. Mountains are located on the northwest side of Taiz governorate and is about 2000 m above sea level. Aqueducts is meant by the valleys where the water is held permanently throughout the year such as Nobaqe, Rasan valleys. The climate where there is the mountain and highland is predominately a cold climate with mild winters in winter and warm to relatively warm in the summer. The abundance of vegetation and variety of the most important trees are available in the province of Samar, *Frangula alnus*, *Acacia nilotica* and *Ziziphus spina*-*christi*, *Acacia drepanolobium*, *Acacia ehrenbergiana*, *Tunb, Ficus benjamina*, *arabic*-*tree*—*Acacia*, *Salvadora persica*, *Tamarix aphylla*, *Cactaceae* and other medical plants some weeds and small plants. Animals and birds: There are many species of wild animals and the most important of these animals hyenas, foxes, tigers, lions, *Lycaon pictus*, rabbits, hedgehogs.

**Fig. 1:**
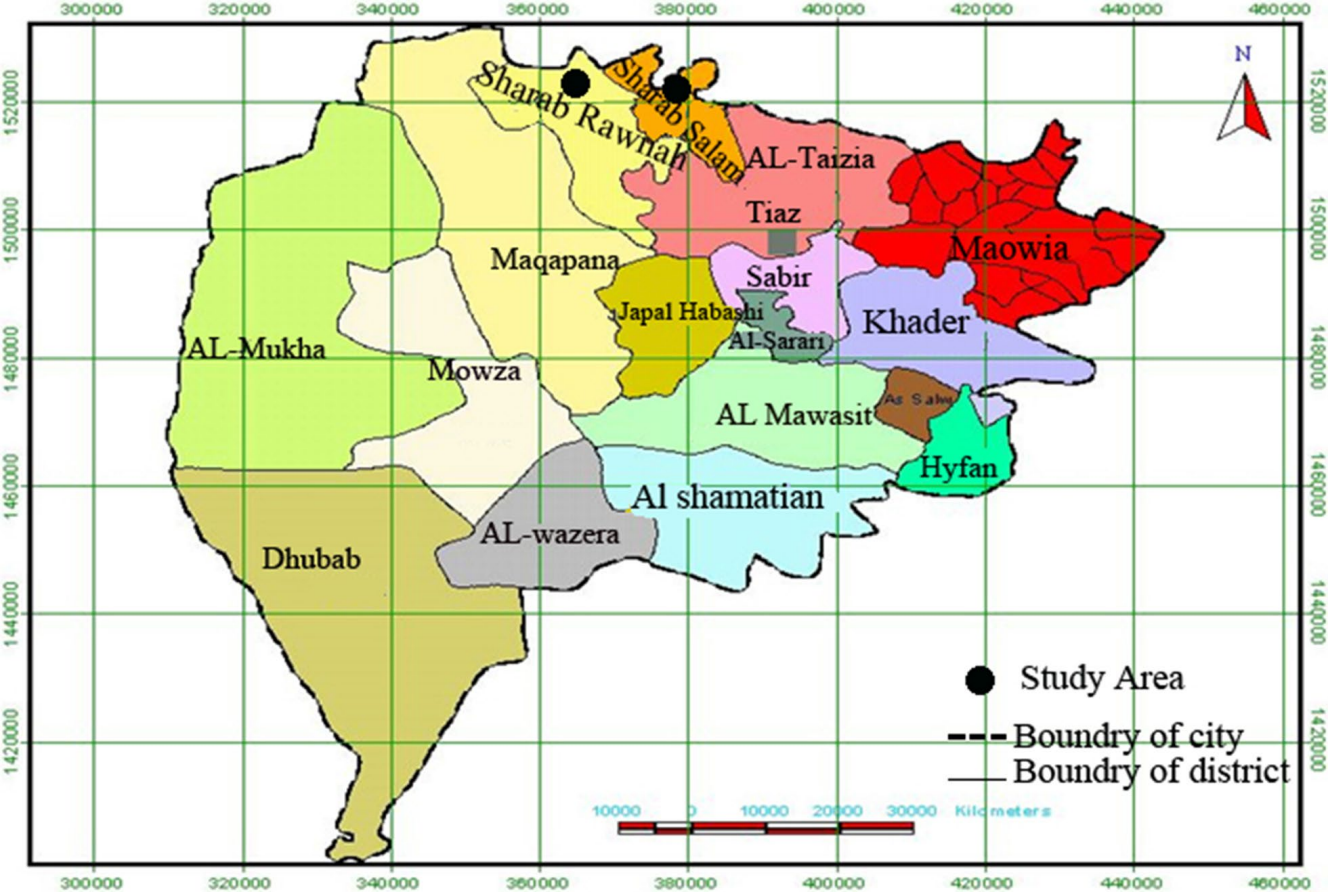
Map of Taiz Governorate, Yemen, shows Shara’b District

The samples were collected from eleven villages located in the above mentioned districts. These villages were chosen for collection from this district. These villages are Banny ziad, Alhosia, Almakhabeer and Nakhla which belong to Shara’b Ar Rownah. Other villages are belong to Shara’b As Salam which include Alamgod, Alzakarer, Banny Sarry, Alafuch, Alshahna, Banny Wahban and Mekhlaf a’ala, as shown in Taiz map (Fig. 1).

### Study population

To assess the prevalence of cutaneous leishmaniasis, 525 villagers ranging between 1 and 60 old years were examined. The survey was conducted of orients as recommended by the World Health Organization (WHO) [13]. Samples of 11 villages in each ecologically homogenous area were considered adequate to evaluate prevalence of Leishmanial infection in an endemic area. Materials used in the present study were collected from eleven villages of Shara’b district. All cases were investigated included: response to the questionnaire containing the required information such as age, sex, education level, house type, defense type, treatment before, drug type and scar sites.

### Cases preparation and blood collection

The samples were collected from April 2012 to October 2013. Cases were collected at Banny Ziad Health Center in Shara’b. Diagnosis was confirmed clinically, as well as by laboratory demonstration of the parasite in the lesions by direct smears using microscopic examination. Tissue scrapings performed with staining the specimen with by Giemsa stain. The lesions cleaned and any eschars or exudates was removed. 1% lidocaine used to decrease bleeding, optimize debridement, and obtain and improve tissue scraping quality. For tissue scrapings, ten scalpel blade was used. Scraping was performed with a pressure that is adequate to obtain exudates, without evoke bleeding. The dermal tissue was spread in a 2–3 cm diameter on a glass slide then fixed briefly with methanol, Giemsa stained, and examined for the presence of amastigotes.

In the second day 10 ml of venous blood were collected from 91 patients as well as from 51 control after an overnight fast and it was positive. The aspirated blood was immediately put into two different test tube (T.T) the other fraction of blood was clotted in plain T.T., then the serum was separated by centrifugation at 1000×*g* and used for the quantitation of the biochemical reaction [14]. This transported by hole freezer immediately to Palestine hospital laboratory and other parts to Alborehee hospital laboratory in Taiz governorate to complete the analysis. Uric acid reagent, MDA reagents [0.5% (W/V) tricholoroacetic acid (BDH), 0.5% (W/V) 2-thiobarbituric acid, 70% tricholoroacetic acid, chloroform (BDH)]. Catalase reagents (ammonium molybdate, hydrogen peroxide, sodium–potassium phosphate buffer), spectrophotometer and waterbath.

## Examination of samples

### Microscopic examination

Lesions were cleaned with ethanol and punctured at the margins of the lesion with a sterile lancet. Smears were made from exudating material, air dried and fixed in methanol. Then they were stained with Giemsa’s stain for examination by light microscopy [13].

### Biochemical tests

#### Measurement of catalase activities

Intracellular catalase enzyme activity was determined according to the modified technique of Goth et al. [15]. A simple method for the determination of serum catalase which included the use of optimized conditions for enzymatic degradation of hydrogen peroxide, spectrophotometric assay of hydrogen peroxide based on formation of its stable complex with ammonium molybdate and measuring the reaction by reading the absorbance by spectrophotometer.

#### Measurement of serum lipid peroxide (MDA) levels

Measurement of serum MDA, secondary product of lipid peroxidation was based on the colorimetric reaction with thiobarbituric acid (TBA). The molar extinction coefficient of MDA is 1.56 × 105 M/cm and the results were expressed as nM of MDA/ml [16].

### Determination of uric acid

Determination of uric acid was by reaction with uricase. The formed H_2_O_2_ reacts under catalysis of peroxidase with 3,5-dichloro-hydroxybenzene-sulfonic acid (DCHBS) and 4-aminophenazone 9 PAP0 to give red–violet quinone-monoimine as indicator [17]. Reaction principle

$$ {\text{Uric acid O}}_{ 2} +  {\text{2H}}_{ 2} {\text{O}}\mathop{\longrightarrow}\limits^{\text{uricase}}{\text{allantion}} + \text{{CO}}_{ 2} + {\text{ H}}_{ 2} {\text{O}}_{ 2} $$

$$ 2 {\text{H}}_{ 2} {\text{O}}_{ 2} + {\text{DCHBS}} + {\text{PAP}}\mathop{\longrightarrow}\limits^{\text{Peroxidase}}{\text{quinone-monoimine}} + {\text{HCL}} + {\text{4H}}_{ 2} {\text{O}} $$

### Statistical analysis

The statistical package for social sciences (SPSS) program on the computer was used. Results were expressed as mean ± stander error of mean (SEM). The data were analyzed by linear regression, paired t test, and two-ways analysis of variances (ANOVA) which was applied for the comparison among different groups. The level of significance was taken as the 0.05.

## Results

### Epidemiological study

#### Prevalence of cutaneous leishmaniasis infection

**Fig. 2:**
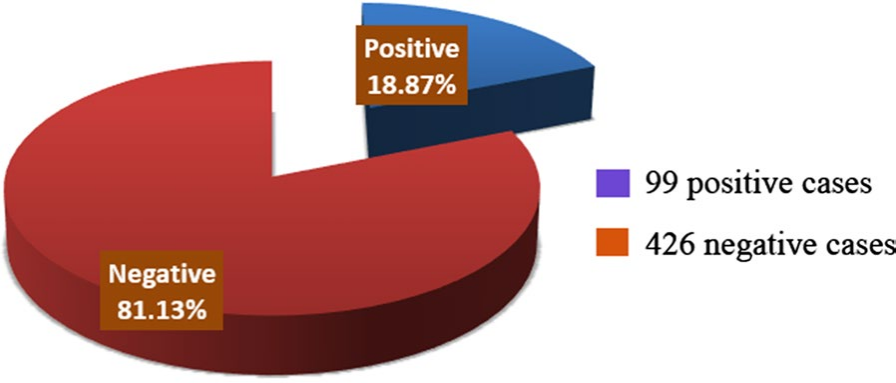
Prevalence of CL infection in Shara’b district, Taiz, Yemen

**Fig. 3:**
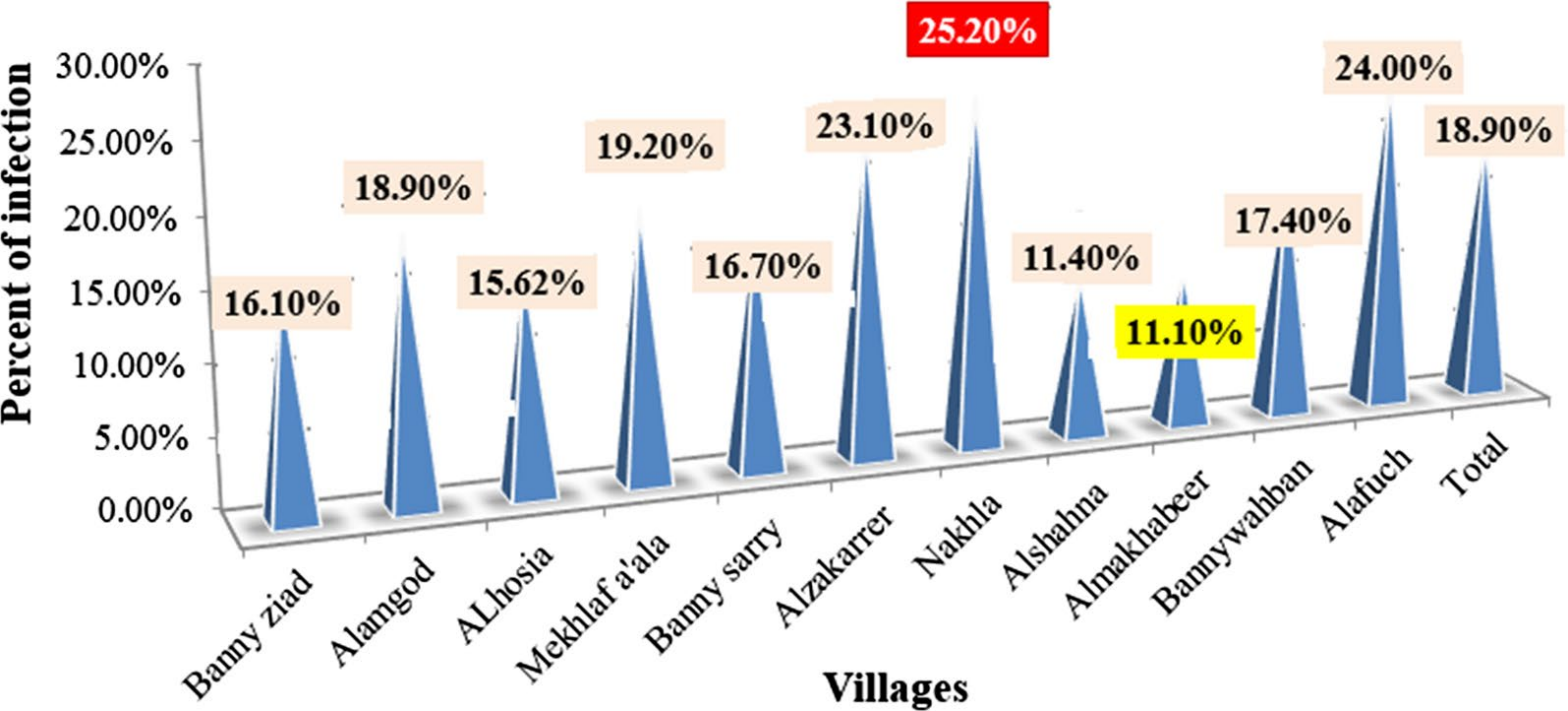
Percent of distribution *Leishmania* infection in villages

Among 525 of a total cases studied, the percent of infected cases are 18.87% and not infected are 81.13% (Fig. 2). Most prevalence rate which were positive collected from Shara’b district was in Nakhla with percent to (25.2%), and the lowest percent in Almakhabeer (11.10%) (Fig. 3).

According to sex, the highest prevalence of CL infection were recorded in males (19.3%) with account (47) and the negative (196) from total male (243) examined. Whereas the lowest prevalence in females with account (52) with a percent of (18.40%) and a negative number (230) from total females (282) cases (Fig. 3; Table 1).

**Table 1:** Rate of CL infection in relation to sex

Sex	No. examined	Overall infection		Odds ratio	(95% CI)	P value
		No.	%			
Male	243	47	19.3	0.011	0.833–0.813	0.044*
Female	282	52	18.4			

* Significant (P < 0.05)

#### Prevalence of CL infection according to the sex and age

**Fig. 4:**
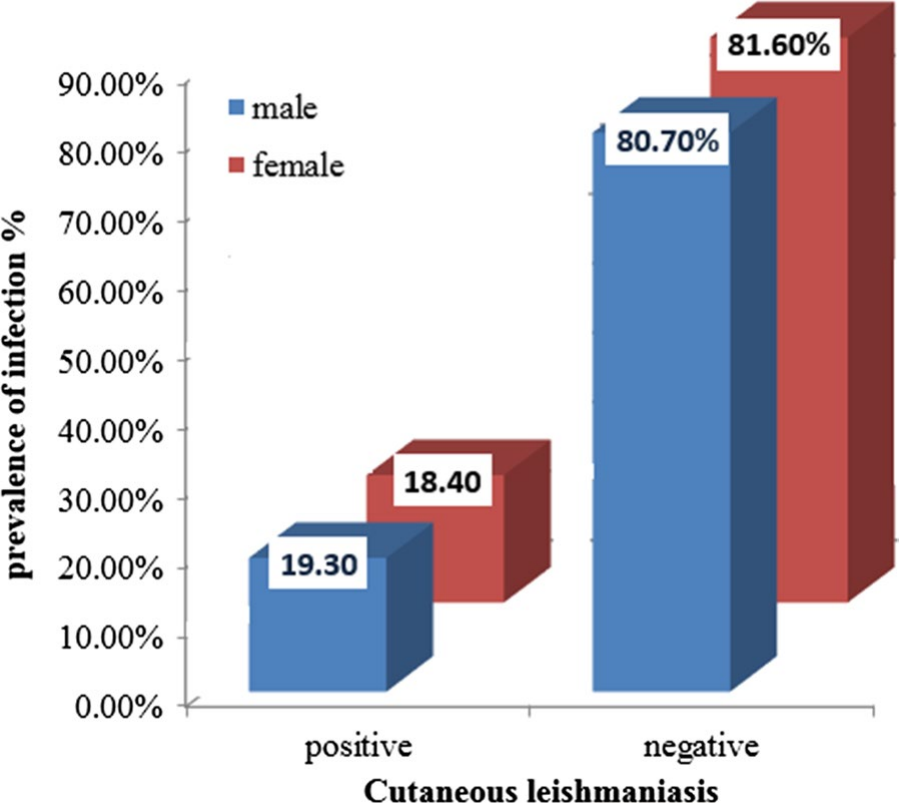
Distribution of cutaneous leishmaniasis among examined cases, according to sex

The distribution of 525 skin scraping samples infection (19.3% males and 18.4% females) shown in Fig. 4. In relation to sex, the prevalence of infection in males (19.3%) was slightly higher than in females (18.4%) there is high significant difference (P < 0.05) Table 1.

In relation to cutaneous leishmaniasis infection among cases examined, according to age and sex groups in Shara’b, district, there was high significant (P < 0.05) between age group 1–15. The males is high 22.7% than in females 18.4% and also there was high significant (P < 0.05) in group 46–60 the high prevalence were in females group 23.5% than in males group 13.6% (Table 2). The rate of Cutaneous leishmaniasis infection in relation to age groups, the age group (1–15) has high significant (OR = 0.458, P < 0.05) than other groups (Table 2).

**Table 2:** Prevalence of CL infection among cases examined, in relation to age and sex groups

Age group	Gender									
	Male			Female			P value	Odds ratio	(95% CI)	Overall infection P value
	No examined	No +ve	% +ve	No examined	No +ve	% +ve				
1–15	88	20	22.7	114	21	18.4	0.013*	0.458	0.943–0.933	0.024*
16–30	90	16	17.8	86	16	18.6	0.373^n^			
31–45	43	8	18.6	65	11	16.9	0.410^n^			
46–60	22	3	13.6	17	4	23.5	0.002*			
Total	243	47	19.3	282	52	18.4	0.555^n^			

^n^non-significant

* Significant difference at (0.05)

#### Types and distribution of lesions

**Fig. 5:**
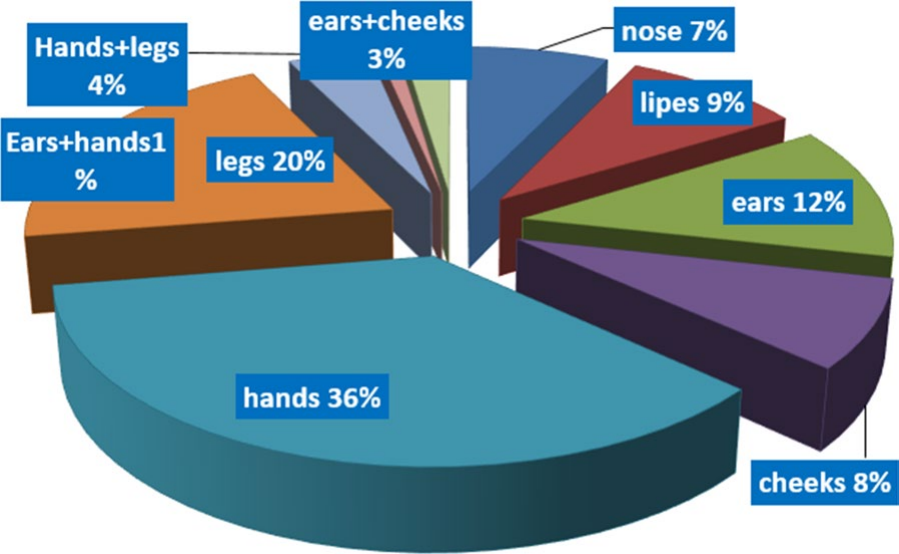
Distribution of lesions on various parts of patients infected with CL

**Fig. 6:**
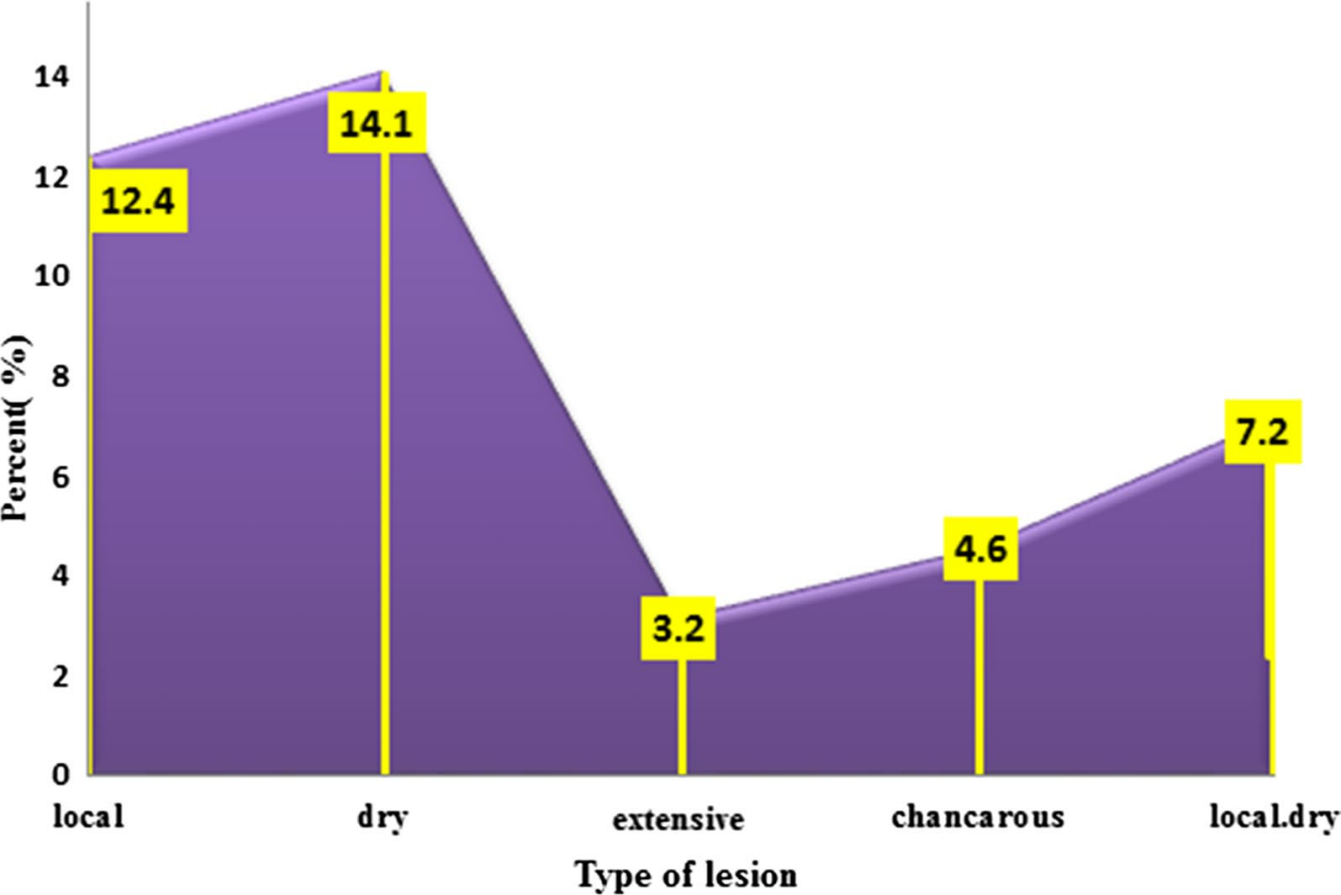
Type of scar on body description infection with CL

Distribution of lesions on various parts on various parts of body in patients shows the highest percent of infection was in hands (36%), and the lowest on the ear and hands (1%) (Fig. 5). In relation to type of scars on body description in infection with cutaneous leishmaniasis in this study the dry scars have were the highest percentage (14%), and the lowest percent with an extensive scars (3.2%) (Fig. 6).

**Fig. 7:**
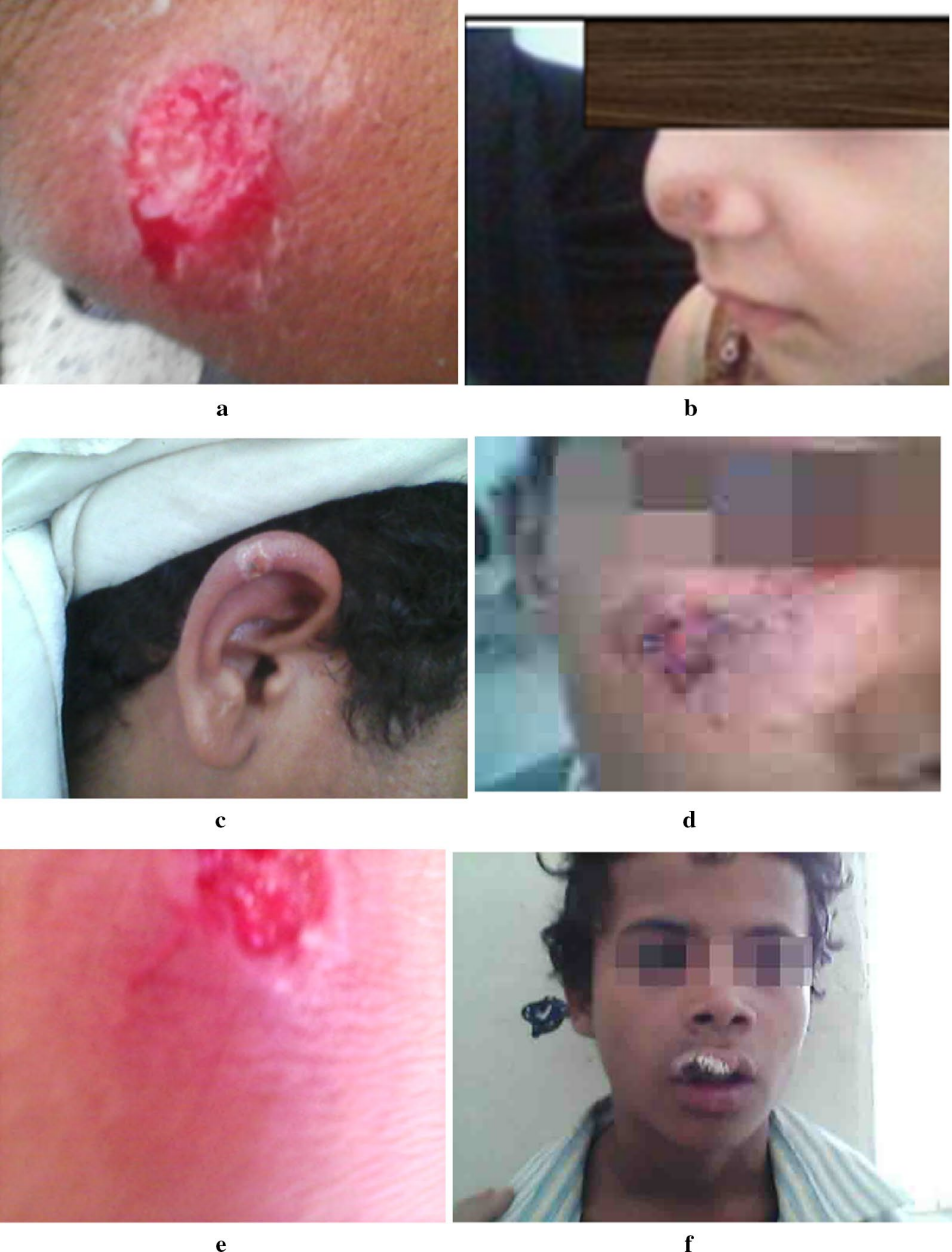
Pictures of lesions in patients with CL. **a** Typical lesion sever inflammatory. **b** Dry lesion on nose. **c** Local and dry lesion on ear. **d** Extensive lesion on cheek. **e** Dry lesion after skin scraping. **f** Dry lesion on upper lip

**Fig. 8:**
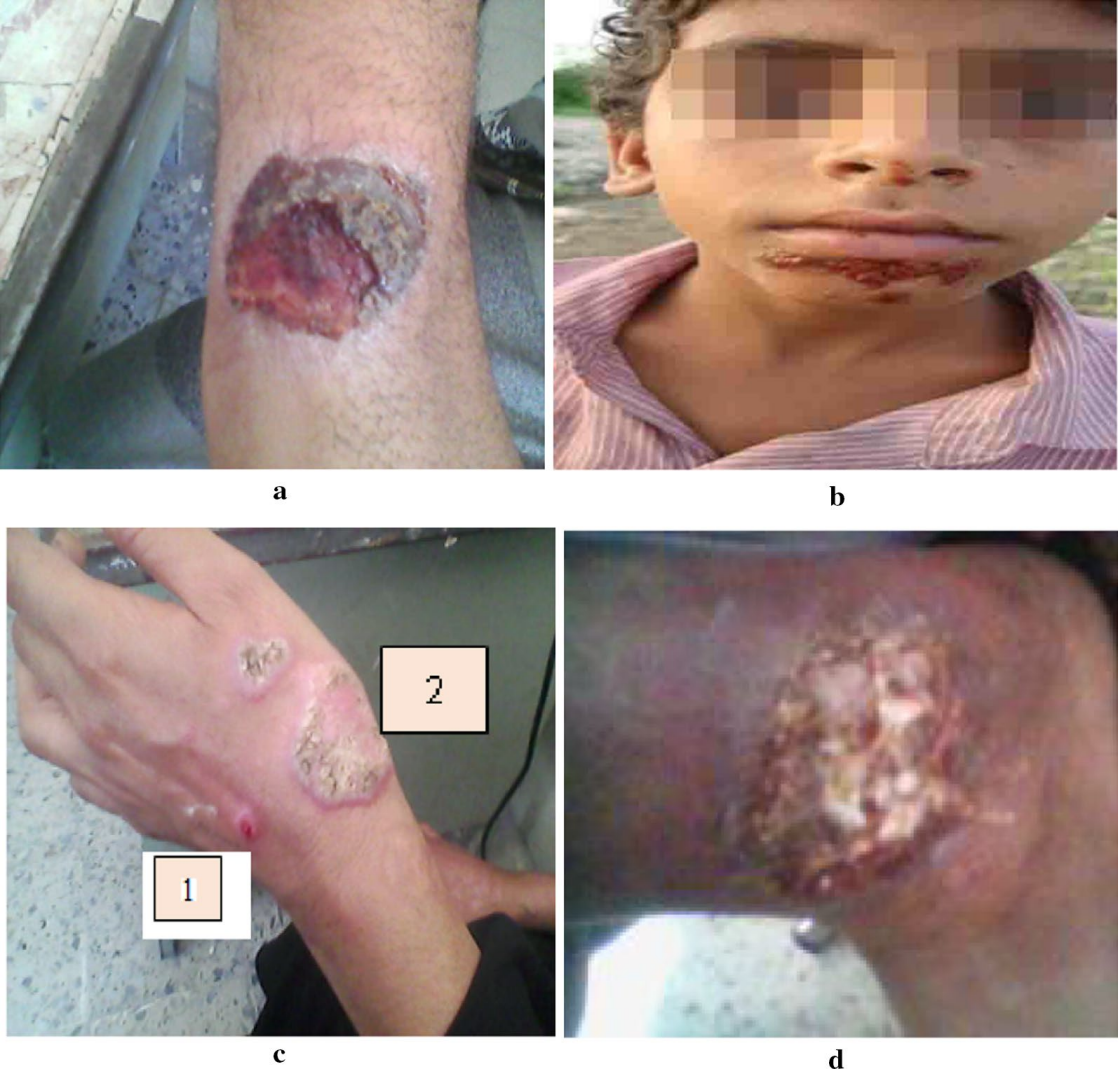
Pictures of lesions in patients with CL. **a** Large lesion on right hand. **b** Lesion under lower lip.** c** *1* Dry lesion and *2* fungi infection.** d** Chancrous lesion on left leg

Also, in relation to the type of scars single lesions were observed in most of the patients. Figures 7 and 8 showed pictures of lesions, of patients with CL in Shara’b, district (a) Typical lesion sever inflammatory reaction, (b) dry lesion on nose, (c) local and dry lesion on right ear, (d) extensive lesion on cheek, (e) dry lesion after skin scraping, (f) dry lesion on upper lip, (g) large lesion on right hand, (h) mucocutaneous infection in mucose of nose with more lesions under lower lip (I); 1 dry lesion; (j) cancerous lesion. In relation to distribution of lesions by site of lesions and age groups, in adult group, the highest percent present in hands (91%) and the lowest lesions in the eras (7.1%), but it is opposite in the child group the highest percent in ears (92.9%) and lowest in hands (8.7%) (Table 3). Leishmaniasis is a diseases caused by obligatory and intracellular haemoflagellate protozoan parasites of the genus *Leishmania* (family trypanosomatidae). Human leishmaniasis is a compound disease with numerous clinical forms, which variety from mild self-healing cutaneous lesions to fatal visceral disease [1].

**Table 3:** Distribution of CL lesions according to site of lesion and age groups

Infected groups		Site of lesions										Total
		No describe	Hands	Legs	Hands and legs	Ears	Cheeks	Lips	Nose	Ear and hands	Ear and cheek	
Child (1–15)	% of total count	1	2	1	1	13.1	9.1	10.1	1	1	2	41.4
	% with site scars	50.0	8.7	20.0	50.0	92.9^a^	37.5	58.8	25.0	25.0	50.0	41.4
	Count	1	2	1	1	13	9	10	1	1	2	41
Adult (16–60)	% of total count	1	21.2	4	1	1	15.2	7.1	3	3	2	58.6
	% with site scars	50	91^a^	80	50	7.1	62	41	75	75	50	58.6
	Count	1	21	4	1	1	15	7	3	3	2	58
	Total	2	23	5	2	14	24	17	4	4	4	99

^a^High percent

Cutaneous leishmaniasis is endemic in the tropics and neotropics. Leishmaniasis was endemic in Yemen [2]. It has been recognized as a public health problem predominated by infection with the highest burden of Leishmaniasis, but has not been fully documented [18]. CL is endemic and most of the cases are registered in Lahg, Abun, Hagga and Sa’adah Taiz Governorates [3]. CL is transmitted by the bite of an infected sand fly. When the parasites enter the polymorph nuclear neutrophils (PML) and the monocyte macrophage cells play an important role in the host defense [5]. These cells are capable to generating a large amounts of extremely toxic molecules, such as reactive oxygen species (ROS), comprise superoxide radicals (O_2_^−^), hydrogen peroxide (H_2_O_2_) and hydroxyl radicals (OH), and reactive nitrogen species (RNS), inclusive nitric oxide (NO). Bogdan C Rolling off Bacteria, parasites and tumor cells motivate macrophages to synthesize considerable amounts of NO which having cytotoxic effects on these activators [19]. ROS and RNS are capable of degrading many biomolecules, including DNA, carbohydrates and proteins. Furthermore, ROS and RNS can assault the polyunsaturated fatty acids of membrane lipids causing lipid peroxidation and the disorder of cell construction and function [4]. Lipid peroxidation is a well-recognized mechanism of cellular injury and is used as a marker of oxidative stress in cells and tissues [6]. Polyunsaturated fatty acid derived that are not stable, can decay hence forming many series of complex products [7]. They are degraded such as carbonyl compound which are plentiful malondialdehyde (MDA) that is widely used as marker of lipid peroxidation [20]. High levels of lipid peroxidation products are accompanied with a variety of chronic diseases with parasitic infections [3]. The serum concentration of MDA was dignified in humans with *cutaneous leishmaniasis* to establish its connection in the pathological mechanism of the disease [9].

To avoid potential oxidative damage there are mechanisms defense systems which are classified as enzymatic [superoxide dismutase (SOD), catalase, glutathione peroxidase (GSH peroxidase), glutathione reductase and GSH reductase] and non-enzymatic (vitamins and uric acid). The estimation of MDA level and antioxidant enzyme activity are main standards in relation to the severity of probable peroxidation, which occur in cell membrane [10]. Anti-oxidant vitamins for instance E, C, and A protect the cells from destruction in contradiction of free oxygen radicals generated consequently of parasites. Antioxidant systems including vitamins have a cellular protective action against oxidative stress subsequent in cell, organ, and tissue damage because of parasitic invasion [11].

#### Rate of cutaneous leishmaniasis infection in relation to certain environmental and social factors

The rate of CL infection in relation to education was showed in Table 4. This table shows that the high rate (54.6%) of infection was seen in cases who have not any level of education, followed by the primary school level (18.2%), secondary school level (18.2%), Diploma (4.0%), Bachelor (5.9%), but it does not give any significant differences (OR = 3.955, P > 0.05).

**Table 4:** Rate of cutaneous leishmaniasis infection in relation to certain social factors

Parameter	No. examined	Overall infection		Odds ratio	(95% CI)	P value
		No.	%			
Primary	86	18	18.2	3.955	0.443–0.424	0.412^n^
Secondary	81	18	18.2			
Diploma	22	4	4.0			
Bachelor	14	5	5.1			
Total	322	54	54.6			

*n* no significant P > 0.05

In relation to certain environmental conditions and the rate of CL infection, Table 5 showed that there was a significant correlation (OR = 0.002, P < 0.05) between infection and animals found in houses, which have no protective defenses. Also, our results show significant correlation between infection and types of residents (OR = 0.035, P < 0.05). The high rate of infection was (19.8%) in populist building than of new building (17.6%) that bullied with mud, cracks and dampness in Table 5. Also, it showed a significant correlation between infection and sitting on the first floor of the house (P < 0.05, OR = 5.50) (Table 6).

**Table 5:** Effect of certain environmental and others social factors on infection with CL

Parameter	No. examined	Over all infection		Odds ratio	(95% CI)	P value
		No.	%			
Residents type						
Populist	346	65	12.38	0.035	0.985–0.456	0.040*
New	179	34	6.47			
Floor type						
First floor	270	61	51.42	5.50	0.778–0.066	0.030*
Second floor	237	34	45.14			
Third floor	18	4	3.43			
House protection						
Found protection	294	55	10.4	0.010	1.00–0.922	0.040*
Not found	231	44	8.38			
Animals found						
Found	314	59	11.23	0.002	1.00–0.524	0.023*
Not found	211	40	7.6			

* Significant P < 0.05

**Table 6:** Levels of maloniadealdhyde (MDA), catalase (Cat.) and uric acid (UA) of patients and healthy control

Test	Number	Mean ± SEM	P value
UA mg/l	Patient (99) Control (51)	Patient 8.5 ± 0.27 Control 5.6 ± 0.17	<0.001*
Cat. kU/l	Patient (99) Control (51)	Patients 72.5 ± 4.5 Control 83.1 ± 4.9	0.115^n^
MDA nM/ml	Patient (99) Control (51)	Patients 3.40 ± 0.06 Control 1.38 ± 0.07	<0.001*

*n* no significant P > 0.05

* Significant P < 0.05

### Free radical scavengers

#### Enzymatic scavenger’s catalase

The mean of catalase activity in the present study decrease (72.53 ± 4.5 K**/**Ul), but this value did not reach a statistically significant in the patient as compared to control (83.11 ± 4.91 K/Ul) in Table 6. In relation to age (Table 7) and sex effects (Table 8) on catalase levels among patients groups and control groups (Table 9) show no significant effect of age on catalase levels among patient and control groups.

**Table 7:** Serum levels of maloniadealdhyde (MDA), catalase (Cat.) and uric acid (UA) in relation to age groups of patients infected with CL

Groups	N	Mean ± SEM	P value
UA mg/l			
1–15	28	8.62 ± 0.42	0.402^n^
16–30	38	8.93 ± 0.50	
31–45	28	7.77 ± 0.50	
45–60	5	8.28 ± 1.49	
Total	99	8.4892 ± 0.27	
Cat. kU/l			
1–15	28	81.96 ± 9.7	0.828^n^
16–30	38	85.34 ± 7.9	
31–45	28	79.49 ± 8.9	
45–60	5	103.11 ± 27. 5	
Total	99	83.43 ± 4.9	
MDA nM/ml			
1–15	28	3.49 ± 0.13	0.139^n^
16–30	38	3.49 ± 0.09	
31–45	28	3.23 ± 0.12	
45–60	5	2.92 ± 0.30	
Total	99	3.39 ± 0.06	

*n* no significant P > 0.05

**Table 8:** Effect of sex on catalase among patients and control groups

Sex	Catalase				
	No.	Mean ± SEM	CI 95%	χ^2^	P value
Males patients	47	73.100 ± 0.021	0.439	39.0	0.561^n^
Females patients	52	72.29 ± 0.014	0.539	44.0	0.461^n^
Males control	27	83.1 ± 0.49	0.0.477	138	0.523^n^
Females control	24	80.1 ± 0.32	0.677	140	0.323^n^

*n* no significant P > 0.05

**Table 9:** Effect of age on uric acid levels among patients and control

Age group	Uric acid		
	χ^2^	OR	P value
Patients	3900.187	692.471	0.363^n^
Control	1063.91	293.908	0.182^n^

*n* no SIGNIFICANT P value >0.05

#### Non enzymatic scavenger uric acid

Our study shows that uric acid level has high significant (P < 0.001) increase in patients groups than those of control groups (Table 7).

In relation to the effect of age on uric acid, Table 10 shows that there is no significant increase of serum levels of uric acid and catalase in age groups of patients infected with CL. Table 11 shows that there is significant (P < 0.05) increase of uric acid in male than in female comparing to healthy groups.

**Table 10:** Effect of age on catalase levels among patient and control groups

Age group	Catalase		
	χ^2^	OR	P value
Patients	40.5	0.0512	0.213^n^
Control	63.48	0.033	0.588^n^

*n* no significant P > 0.05

**Table 11:** Effect of sex on uric acid level among infected group with CL and control group

Test group	Sex	Number	Mean and SEM	P value
Uric acid	Patients males	47	9.70 ± 0.35	0.050*
	Control males	27	6.62 ± 0.40	0.527^n^
	Patients females	52	8.10 ± 0.44	0.511^n^
	Control females	24	6.91 ± 0.41	0.492^n^

*n* no significant P > 0.05

* Significant P < 0.05

## Discussion

### Cutaneous leishmaniasis

According to results obtained, among 525 of total cases was studied, 99 cases with a percentage of 18.87% was infected with CL and 426 cases not infected with percent of 81.13%. The prevalence of infection was higher in males (19.3%) than in females (18.40%). The same results were reported by AL-Jawabreh et al. [20], and Silva et al. [21].

Cutaneous leishmaniasis (CL) is a social problem in the tropics and subtropics [22] and the North Western [23]. CL reported in the most decade in Yemen, mostly among young children, including the Governorates of Sana’a, Taiz, Ibb, Alhodeidah, Hajjah, Damar, Sa’adah, Al-Mahweet, Ma’arab, and Aljawf [24]. Differences were seen between males and females, overly more males were infected. This is probably happened due to the cultural habits of these areas, as they are exposing themselves to sand flies bites [25] there was a slightly increased positive percent of infection in females group. This may be due to the females staying in these epidemic villages with sand flies all the time to make all jobs including cultures activities. The highest percent of infection was in Nakhla (25.2%) due to the geographical site which was near mountains and has water stream flow all year and abundant of fresh water holes which provide sand flies with a suitable environment to complete its life cycle and increase agriculture activities. Similar results found by Al-Qubaty [3] in the Western area of Yemen. The low percent of infection in Almakhabeer (11.10%) was due to the decrease water sources and decreased of agricultural activity and the geographical site of these village have many mountains, which play in distribution of sand fly [26] that may explain the lowest rate of infection.

The most infected cases in the present study were increased from April 2012 to October 2013. The climate has many subtropical features; the mean annual temperature lying between 20 and 30 °C with little seasonal variation. The relative humidity ranging between (40–60%) in the Western Yemen with relatively high rainfall in summer and its sub humid warm-temperature climate with a distinct dry period during the winter months. The annual rainfall from approximately 800–1200 mm, and most of this falls from April to October. The middle heights are well watered by perennial streams (Wadies) and small irrigation channels, temporary streams and pools are plentiful during the rainy seasons [27]. The majority of the populations are engaged in agriculture near their houses where sand flies are found, which the primary source of income is, so this climate may explain why the leishmanias is has wide spreads in this village. These results show that the highest infection is significant (P < 0.05) among males aged group of (1–15), but not in females of the same aged group. This may be due to the increased activity of males aged than females during this age. Wearers the significant (P < 0.05) increased is observed in females aged (46–60 years) than males that may due to low immunity of females in this age.

The reasons of high significant and higher prevalence rate (OR = 0.458, P < 0.05) in younger age is probably due to the fact that they have poorly developed an immune system. They cannot prevent themselves from bites of sand flies and do not have cultural knowledge of defending themselves. Similar results and discussion were observed by [21]. As most of the people residing in the endemic areas are not aware of disease, public health education is of great importance. They were not taught to change their sleeping habit to avoid cracks, dampness in their houses, and did not to keep the surrounding free of sand fly. Although animals seem to be reservoirs, this possibility should not be ruled out by carrying our surveillance in the most likely domestic and wild animals. Similar results and discussion found by [26].

These results showed that there is a significant correlation (OR = 0.002, P < 0.05) between infection and animals found in houses, which have not protective defenses (OR = 0.010, P < 0.05). Human and domestic animals are accidental hosts for any *Leishmania* spp. which are maintained in cycles between wild animals and sand flies [28].

*Leishmania infantum*, *Leishmania peruviana* and possibly other species can be found in dogs, increasing the risk of transmission to people. [29] Other domesticate animals might be involved as secondary maintenance hosts. *Leishmania donovani* and *Leishmania tropica* are adapted to humans, but animals can also be infected occasionally [28]. The occurrence of this outbreak of Zoonotic cutaneous leishmaniasis in the district seems to be the results of construction of buildings near colonies of rodents and also travelling to the other infected foci of Shara’b. This result was the same by Alkhavan et al. [30]. This study shows a significant correlation (P < 0.05) between infection and types of residents. The high percent of the infection is 19.8% in populist building than of new building (17.6%) that bullied with mud, cracks and dampness which makes suitable environmental for sand flies colonies. Also, this result was the same by Alkhavan et al. [30]. The present study shows a significant correlation (OR = 5.50, P < 0.05) between infection and sitting in the floor house. The same results were found by Surendrana et al. [26]. They found that floors and plinths of houses, soil at the edges of heaps of refuse, and soil at the bases of stone walls are good breeding sites for the sand flies.

The results of this study show that the sites of CL distribution among the infected cases are related to age. The highest percent of lesions among adults were present in hand, leg, cheek and nose respectively, but the highest lesions of children lesions were present in ears, nose, and legs respectively. These results were in agreement with the results of AL-Jawabreh et al. [20] who found that in children the head was a more frequently infected area (61.3%) than other body sites of children, whereas the limbs were more involved in adults (78.3%). The distribution of lesions in the head area had a certain pattern with lesions more often appearing on the cheek (29.6% of 115 lesions) than on the nose (23.5%), the forehead (14.8%) or the chin (13.0%). Our results show that the infection of hands is the highest with (36%). The lesions were increased in hand than in the others parts because hands are for the most part that might be exposed to the bites. Similar results were observed by Ullah et al. [31].

In the present study, the single lesions are observed in most of the patients, which is supported by Parks [32]. The cause of differences of scars type maybe due to the variation of the vectors. The same discussion explained by Kharfi [25], present study does not cover the vector. Cutaneous leishmaniasis in Shara’b is caused by *Leishmania*. The vectors are sand flies of the genus *Phlebotomies*, there was not study made about the species that cause the disease. The incubation period ranges from weeks to months. Present study shows some lesions appeared typically on exposed areas of the body where inoculation occurs. The same scars have been founded by Mandel et al. [33]; Mings et al. [34]. Lesions appear as small nodules, or round with raised margins and a granulating center with yellowish exudates which increase in size and eventually ulcerate, that depend on parasite, host, and sand fly factors; dose or route of inoculation; and the maintenance of macrophages in an inert, deactivated state [35]. The morphologic characteristics depend on the complex interactions between the virulent characteristics of the infecting *Leishmania* sp. and T-cell mediated immune responses of its human host [36].

### Changes in lipid peroxidation and some free radicals scavengers in patients and control

#### Serum lipid peroxidation malondialdehyde (MDA) levels

Highly reactive oxygen species free radicals (ROS) have been indicated in the pathogenesis of various parasitic infections including *Leishmania* [37], *Plasmodium falciparum* (Kumar and Das 1999). *Ascaris lumbricoides* (Kilic et al. 2003), *Toxoplasma gondii* [38] *Trypanosoma cruzi* [39]. Lipid peroxidation is an ongoing physiological process, but several lines of evidence have suggested an important role for peroxidation in the pathogenesis of several parasitic diseases [40]. Lipid peroxidation is caused by ROS results in the disarrangement and ultimately, disruption of cell membranes, which leads to necrotic and cell death. The significant higher increased of serum MDA (P < 0.001) in CL patients, as comparing to control level of MDA, may suggest that the overproduction of ROS and RNS results in oxidative stress, and the acceleration of lipid peroxidation in CL patients, resulting from altered enzymatic antioxidant activities may be considered as an indication of cell injury caused by *Leishmania*.

Increased levels of MDA in serum of infected animals is related to the host defense against parasitic infections. The similar results were observed by Kocyigit et al. [4] and Serarslan et al. [41]. They found significant increase of serum MDA and NO^·^ in CL patients, as compared to their control and by Ozbilge et al. [42] who showed significant increase in LPO, superoxide dismutase peroxidation (SOP), glutathione and decrease catalase activity levels in patients with active CL than those of healthy control. Our result reveals that no effect of age and sex on the mean MDA levels in patients groups with CL and controls groups. Similar findings were reported by Quassim [43] and AL-Shamiri [44] as they found no significant changing in MDA levels among age groups of control and patients groups which disagree with the results of Hassan [45] who observed a significant increase in plasma MDA in the age of control group of (27–44 years) as compared to older age groups (45–58 years) and she suggested that this increased in MDA level to decreased SOD scavenger. From the previous speculation and the present observation, it might be postulated that the high serum MDA values in CL reflects an increased lipid peroxidation initiated by reaction of free radicals with poly unsaturated fatty acids in biological membranes. More over rapid production of oxygen free radicals depletes the protective antioxidant and enzymes.

#### Free radical scavengers

##### Enzymatic scavenger’s catalase

In the present study there was no significant decrease of catalase activity in patient as comparing to control and these result was in disagreement with result of Erel et al. [46]; Kocyigit et al. [4] they found that there was a significant decrease of mean catalase activity which level and increased MDA levels in patient with cutaneous leishmaniasis as compared to control. The mechanism of decrease catalase activity was due to that serum catalase activity can alter H_2_O_2_-dependent reactions and in the other site resistant the parasite to H_2_O_2_ which causes a consumed catalase serum.

The non-significant decrease of catalase activity in our study may happen due to the fact that the parasite itself is protected to some extent against toxic oxygen metabolites. As discussed earlier, by Murray [47]. Amastigotes appear to contain catalase and superoxide dismutase although leishmania is poorly endowed in glutathione peroxidase [47] a novel reducing agent specific to trypanosomes has been described, which may serve to mop up hydrogen peroxide evolved during the respiratory burst [48]. This happens, maybe due to the method that we used in the present study, as well.

The large variation in age groups of patients in the present study may explain a decrease of catalase activity. Different result in catalase activates was recorded by Niwa et al. [49]. They found that catalase, glutathione peroxidase and d-glucose-6-phosphate dehydrogenase, was significantly higher in younger adults than in elderly individuals. The basic levels of three other H_2_O_2_ scavenging enzyme activities were found to be decreased in leukocytes of elderly adults in comparing with young adults. This results shows that there is any significant effect of age and sex on catalase levels among controls and patients groups. Our study of Yemeni individual has not been studied yet and there were not a number of normal values of serum catalase activity.

##### Non enzymatic scavengers

Uric acid: Uric acid is an important contributor to total antioxidant capacity; it provides a significant antioxidant defense against nitration by proxy nitrite. It has an important role as an oxidative stress marker and a potential therapeutic role as an antioxidant [50].

This study shows that there is a high significant (P < 0.001) increase of the uric acid level in patients groups than those of controls. The same result was reported by Frederico et al. [51]. This increase in uric acid level which may refer to the physiological activity and the influence of destroyed or catabolism [52, 53]. Increased level of uric acid may contribute much more to scavenging of free radicals. This may support the powerful antioxidant role of uric acid in scavenging singlet oxygen and other free radicals [54] and [55]. Uric acid may act as a defense mechanism against oxidative stress, or uric acid acting as a pro-oxidant and contributing to the damage caused in these diseases [56, 57]. Uric acid is released from tissues that are short of oxygen and elevated uric acid levels may an important part of acclimatization to high altitude [58, 59]. There is a significant increase due to the effect of sex on uric acid in male than female this may due to its scavenger activities.

## Conclusions

We conclude from this study that there was a high spread of CL and there were high prevalence rates of cutaneous leishmaniasis in Shara’b District, Taiz, Yemen, The prevalence of CL was highly positive in Nakhla village. The rate of infection among males was higher than females. There was an association between the infection and age group. The patient who had cutaneous leishmaniasis has many changes in some biochemical levels. This study provides a clear indication of the role of MDA as an early biochemical marker of peroxidation damage occurring during cutaneous leishmaniasis. Increased uric acid, and catalase activity was provided of free radical scavengers.
